# External quality assessment of malaria microscopy diagnosis in selected health facilities in Western Oromia, Ethiopia

**DOI:** 10.1186/s12936-018-2386-2

**Published:** 2018-06-18

**Authors:** Getachew Sori, Olifan Zewdie, Geletta Tadele, Abdi Samuel

**Affiliations:** 1 Nekemte Regional Laboratory, Nekemte, Ethiopia; 2 Department of Medical Laboratory Sciences, College of Medical and Health Sciences, Wollega University, P.O. Box: 395, Nekemte, Ethiopia

**Keywords:** External quality assessment, Malaria microscopy, Western Oromia

## Abstract

**Background:**

Accurate early diagnosis and prompt treatment are one of the key strategies to control and prevent malaria disease. External quality assessment is the most effective method for evaluation of the quality of malaria microscopy diagnosis. The aim of this study was to assess the quality of malaria microscopy diagnosis and its associated factors in selected public health facility laboratories in East Wollega Zone, Western Ethiopia.

**Methods:**

Facility-based cross-sectional study design was conducted in 30 randomly selected public health facility laboratories from November 2014 to January 2015 in East Wollega Zone, Western Ethiopia. Ten validated stained malaria panel slides with known *Plasmodium* species, developmental stage and parasite density were distributed. Data were captured; cleaned and analyzed using SPSS version 20 statistical software-multivariate logistic regressions and the agreement in reading between the peripheral diagnostic centers and the reference laboratory were done using kappa statistics.

**Results:**

A total of 30 health facility laboratories were involved in the study and the overall quality of malaria microscopy diagnosis was poor (62.3%). The associated predictors of quality in this diagnosis were in-service training [(AOR = 16, 95% CI (1.3, 1.96)], smearing quality [(AOR = 24, 95% CI (1.8, 3.13)], staining quality [(AOR = 15, 95% CI (2.35, 8.61), parasite detection [(AOR = 9, 95% CI (1.1, 8.52)] and identification skills [(AOR = 8.6, 95% CI (1.21, 1.63)]. Eighteen (60%) of health facility laboratories had in-service trained laboratory professionals on malaria microscopy diagnosis.

**Conclusion:**

Overall quality of malaria microscopy diagnosis was poor and a significant gap in this service was observed that could impact on its diagnostic services.

## Background

Malaria remains a major global public health challenge. In 2016, 91 countries reported a total of 216 million cases of malaria, an increase of 5 million cases over the previous year. The global tally of malaria deaths reached 445,000 deaths, about the same number reported in 2015. The WHO African Region continues to account for about 90% of malaria cases and deaths worldwide. Fifteen countries—all but one in sub-Saharan Africa—carry 80% of the global malaria burden [1]. The malaria parasite is transmitted from an infected person to another by the bite of a female Anopheles mosquito. Transmission can occur only after the parasite has been inside the mosquito for at least a week [2].

Rapid and effective malaria diagnosis not only alleviates the suffering but also decreases community transmission. The nonspecific nature of clinical signs and symptoms of malaria may result in over-treatment of malaria or non-treatment of other diseases in malaria-endemic areas, and misdiagnosis in non-endemic areas [3]. In the laboratory, malaria is diagnosed using different techniques such as conventional microscopic diagnosis by staining thin and thick peripheral blood smears, and other concentration techniques, such as quantitative buffy coat (QBC) method, rapid diagnostic tests (RDTs) and molecular diagnostic methods [2].

The World Health Organization (WHO) recommends cross-checking of blood slides. A sample of routine blood slides is sent to the reference laboratory, where it is checked for accuracy. External quality assessment (EQA) programmes is an alternative approach. In such programmes, the reference laboratory sends stained blood film samples to the peripheral laboratories, which assess them and submit a report, after which they are given feedback about the correct results and their own performance [4].

The high sensitivity of diagnosis in malaria-endemic areas is particularly important for the most vulnerable population groups, such as young children and non-immune populations, in whom the disease can rapidly be fatal [5]. Malaria control requires a functional laboratory set-up with quality diagnostic service, trained professionals and microscopists to halt the burden. This work requires concentration in order to assess the quality of blood film malaria microscopy for the detection of *Plasmodium* species by proficient testing, blinded slide rechecking using checklist to identify any gaps in providing malaria services in selected health facility laboratories in the Western Oromia, Ethiopia.

## Methods

### Study area and period

This study was conducted in East Wollega Zone of the Oromia National Regional State, Western Oromia, Ethiopia, from November 2014 to January 2015. The temperature was 10.9–33.9 °C, annual rainfall was 1000–2400 mm and topography was 4.91% high land, 53.17% mid land, and 41.92% low land. The zone has 61 public health facilities, one of which is a referral hospital, one a district hospital, and 59 health centres [6]. All health facility laboratories provide malaria microscopy services, except Gaba Jimata health centre in Gida Ayana Woreda. The study was conducted in 30 health facility laboratories that were randomly selected from 60 malaria microscopy providing public health facilities.

### Study procedures

Verified stained panel slides were distributed to 30 laboratory professionals from selected public health facilities and the results were collected. The validated panel slides were distributed for reading to all selected malaria microscopy health facility laboratory professionals. The panel slides included 10 stained samples with different composition of *Plasmodium* species, the developmental stage of the parasite and parasite density as recommended by national guidelines [7]. The time allowed for reading 10 slides was 50–70 min according to national guidelines recommend examination of 100/HPF (High Power Field) (100 × objective). EQA of malaria microscopy is an essential requirement for malaria care in a district. The focus of EQA is on the identification of laboratories where there may be serious problems resulting in poor performance, not on identification of individual slide errors or validation of individual patient diagnosis. It helps to ensure the trust-worthiness of smear results through the following:

#### On-site evaluation

Malaria laboratory activities in all selected malaria microscopy diagnosis in the health facilities were observed and all heads of departments of the respective health facilities were interviewed using WHO-AFRO checklists to obtain a realistic assessment of the overall operational conditions and skills in the laboratory.

#### Blind rechecking

Slides are rechecked for quality of blood film preparation, staining and accuracy of result. Rechecking reflects the true performance of routine diagnostic services at health facility level. For this study, the blood film slides were collected from the selected malaria microscopy providing health facilities, which were selected in accordance with WHO recommendation of a minimum of five positives and five negatives slides per month.

### Data analysis

Data were captured, cleaned and analysed using SPSS version 20 statistical software-multivariate logistic regressions and *P* value of less than 0.05 was considered to be statistically significant. The specificities, sensitivities, positive predictive value, negative predictive value of slide reading by the laboratory professionals were assessed. Agreement in reading between peripheral diagnostic centres and the reference laboratory readings were interpreted using kappa value. Accuracy is defined as the closeness of the measured result to the true value and malaria microscopy was used as the gold standard in the study.

## Results

### Quality of malaria microscopy: panel slides

A total of 300 panel slides were distributed to 30 malaria microscopy diagnosing centres for 30 laboratory personnel. Of the total facilities, 6 (20%) of laboratory professionals scored an excellent agreement with reference reader (kappa = 1.00) on parasite detection and 6 (20%) scored slight agreement (kappa = 0.0–0.2) (Table 1).

**Table 1:** Sensitivity, specificity and agreement of each health facility laboratory professionals with level 1 malaria microscopist on malaria microscopy diagnosis Western Oromia, Ethiopia

Id of HF lab.	Sensitivity %	Specificity %	NPV	PPV	Agreement (%)	Kappa value
Lab 1	88	50	50	88	80	0.4
Lab 2	71	50	20	100	70	0.4
Lab 3	88	50	20	88	80	0.6
Lab 4	75	100	50	100	80	0.5
Lab 5	83	50	25	100	80	0.7
Lab 6	88	100	67	100	90	0.7
Lab 7	88	100	67	100	90	0.7
Lab 8	100	100	100	100	100	1.0
Lab 9	63	100	40	100	70	0.4
Lab 10	63	50	25	83	60	0.1
Lab 11	88	100	67	100	90	0.7
Lab 12	71	100	50	83	80	0.6
Lab 13	100	100	100	100	100	1.0
Lab 14	75	100	50	100	80	0.5
Lab 15	100	100	100	100	100	1.0
Lab 16	63	50	25	83	60	0.1
Lab 17	88	100	67	100	90	0.7
Lab 18	75	100	50	100	80	0.5
Lab 19	63	50	25	83	60	0.1
Lab 20	88	100	67	100	90	0.7
Lab 21	100	100	100	100	100	1.0
Lab 22	63	100	40	100	70	0.4
Lab 23	33	50	20	80	40	0.3
Lab 24	63	100	40	100	70	0.4
Lab 25	63	50	25	83	60	0.1
Lab 26	75	100	50	100	80	0.5
Lab 27	88	100	67	100	90	0.7
Lab 28	50	50	20	80	50	0.0
Lab 29	100	100	100	100	100	1.0
Lab 30	100	100	100	100	100	1.0
	77%	83.3%			78%	0.5

Based on national guideline for evaluation of laboratory professionals on panel slide examination, 1 (3.3%) of laboratory professionals correctly read all positive slides with correct parasite quantification. Twelve (40%) did not try to report parasite density. 17 (56.7%) correctly quantified the parasite density in at least one positive slide which agreed with the reference density established for each slide. Six (20%) of laboratory professionals reported all positive slides as positive and 20 (66.7%) correctly reported all negative slides. Twenty-nine (96.7%) of participants missed species identification in at least one positive slide (Table 2).

**Table 2:** Grading of laboratory performance based on result of panel slides in selected public health facility laboratories Western Oromia, Ethiopia

HF Lab. ID	Positive reported as negative or vice versa (zero points/slide)	Positive reported as positive (three points/slide)	Correct species (three points/slide)	Correct parasite stage (two points/slide)	Correct parasite load (two points/slide)	Negative reported as negative ten points per slide	Cumulative score	Performance
Lab-1	2*0 = 0	21	12	8	8	10	59	Poor
Lab-2	3*0 = 0	15	6	4	6	20	51	Poor
Lab-3	5 × 0 = 0	12	0	0	0	10	22	Poor
Lab-4	2*0 = 0	18	9	6	4	20	57	Poor
Lab-5	4 × 0 = 0	15	6	4	0	10	35	Poor
Lab-6	1 × 0 = 0	21	18	12	4	10	65	Poor
Lab-7	1 × 0 = 0	21	8	8	4	20	61	Poor
Lab-8	No error	24	24	16	16	20	100	Excellent
Lab-9	3 × 0 = 0	15	0	0	0	20	35	Poor
Lab-10	4 × 0 = 0	15	0	0	0	10	25	Poor
Lab-11	1 × 0 = 0	21	21	14	6	20	82	Good
Lab-12	2 × 0 = 0	18	12	8	6	20	64	Poor
Lab-13	No error	24	18	12	6	20	80	Good
Lab-14	2 × 0 = 0	18	3	2	0	20	43	Poor
Lab-15	No error	24	21	14	6	20	85	Good
Lab-16	4 × 0 = 0	15	3	2	0	10	30	Poor
Lab-17	1 × 0 = 0	21	18	12	8	20	79	Good
Lab-18	2 × 0 = 0	18	9	6	4	20	57	Poor
Lab-19	4 × 0 = 0	15	6	4	0	10	35	Poor
Lab-20	1 × 0 = 0	21	18	12	6	20	77	Good
Lab-21	No error	24	21	14	4	20	83	Good
Lab-22	3 × 0 = 0	15	3	2	0	20	40	Poor
Lab-23	5 × 0 = 0	12	6	4	0	10	32	Poor
Lab-24	3 × 0 = 0	15	3	2	0	20	40	Poor
Lab-25	4 × 0 = 0	15	9	6	2	10	42	Poor
Lab-26	2 × 0 = 0	18	9	6	0	20	53	poor
Lab-27	1 × 0 = 0	21	15	10	6	20	72	Poor
Lab-28	5 × 0 = 0	12	0	0	0	10	22	Poor
Lab-29	No error	24	21	14	8	20	87	Good
Lab-30	No error	24	21	14	8	20	87	Good
Overall average points							57	Poor

Of 300 panel slides, 240 positive panel slides were distributed which comprised 90 (37.5%) *Plasmodium falciparum*, 90 (37.5%) *Plasmodium vivax* and 60 (25%) mixed of *P. falciparum* and *P. vivax*. Forty-two (46.7%) and 47 (52.2%) of the slides were correctly detected and identified for *P. falciparum* and *P. vivax*, respectively. Detection error was reported in 33 (36.7%) for *P. falciparum,* 22 (24.5%) for *P. vivax* and 70% *Plasmodium* species identification error from mixed infection. Health facilities that participated in the EQA programme had considerable agreement (kappa = 0.75) with reference reader on malaria detection by microscopy when compared with health facilities that did not participate in the EQA programme (kappa value = 0.31). Comparison between in-service training in malaria detection was higher in trained laboratory professionals (kappa = 0.58) when it was compared with untrained in-service professionals in selected health the facilities (kappa = 0.56) (Table 3).

**Table 3:** Overall sensitivity, specificity and agreement of public health facility laboratory professionals with level 1 malaria microscopist in detecting malaria parasites Western Oromia, Ethiopia

Peripheral laboratory	Sensitivity	Specificity	Agreement %	Kappa value
	77%	83.3%	78	0.50
In-service training				
Trained	81.2	90.9	83.2	0.578
Untrained	64.0	68.7	65	0.56
EQA participation				
Participated	89.4	96.1	90.7	0.746
Not participated	66.9	76.4	68.8	0.3077
Qualification				
B.Sc. degree	82.5	90	84	0.592
Diploma	73.8	82.5	75.5	0.42

### Random blind rechecking

Overall sensitivity and specificity of health facilities in detection and identification of *Plasmodium* species were 78 and 83.7%, respectively. The overall false positive and false negative rates were 98 (24.4%) and 85 (14.4%), respectively and the overall agreement between health facility laboratory and regional laboratory experts on malaria microscopy diagnosis (random blind rechecking) was 82% (kappa = 0.62).

### Professional background and number of laboratory professionals in selected laboratories

The selected health facility laboratories had a total of 53 laboratory professionals, of which 17 (32%) were degree and 36 (68%) were diploma level educated. 17 (56.7%) of health facilities had 2 laboratory professionals and 12 (40%) 1 laboratory professional. Of the laboratory professionals, 39 (73.6%) were trained in malaria microscopy diagnosis. Fifty (94.3%) of the laboratory personnel had service of 2 and more years.

### Factors associated with the quality of malaria microscopy

To refine any confounding factors, a multivariate logistic regression model was used. According to this model, factors such as in-service training, quality of staining and quality of smearing, remained the predictors for quality of malaria microscopy. Trained laboratory professionals on malaria microscopy diagnosis and quality assurance were 16 times more likely to produce quality of malaria microscopy diagnosis than untrained laboratory professionals [(AOR = 16, 95% CI of (1.3–1.96)]. Health facility laboratories preparing good stained blood films were 10 times more likely to harvest good quality in the malaria microscopy diagnosis than poorly staining blood films [(AOR = 15, 95% CI of (2.35, 8.61)]. Preparing good blood films was 24 times more likely in quality of malaria microscopy than poorly performing blood films [(AOR = 24, 95% CI of (1.8, 3.13)] (Table 4).

**Table 4:** Factors associated with quality of malaria microscopy in selected public health facility laboratories Western Oromia, Ethiopia

Variables	Malaria microscopy quality		OR (95%) CI		
	Yes (%)	No (%)	COR	AOR	P-value
EQA participation					
Yes	8 (80%)	2 (20%)	22 (3.1–163)	10 (0.4–2.231)	0.561
No	3 (15%)	17 (85%)	1.00	1.00	
Use of buffered water					
Yes	7 (77.8%)	2 (22.2%)	19 (27–145)	0.1 (0.012–1.07)	0.44
No	4 (19%)	17 (81%)	1.00	1.00	
Internal quality control					
Yes	7 (63.6%)	4 (36.4%)	6 (1.2–34)	1.6 (0.18–141)	0.11
No	4 (21.1%)	15 (78.9%)	1.00	1.00	
Practice staining quality					
Yes	5 (71.4%)	2 (28.6%)	7 (1.07–14.6.)	15 (2.35–18.6)	0.039*
No	6 (26.1%)	17 (73.9%)	1.00	1.00	
In service training					
Yes	10 (55.6%)	8 (44.4%)	13 (1.4–130)	16 (1.3–19.6)	0.041*
No	1 (8.3%)	11 (91.7%)	1.00	1.00	
Qualification					
Diploma	6 (30%)	14 (70%)	2 (0.48–11)	0.4 (0.09–2.05)	0.285
B.Sc.	5 (50%)	5 (50%)	1.00		
Smearing quality					
Yes	7 (70%)	3 (30%)	0.1 (0.19–0.61)	24 (1.8–31.3)	0.037*
No	4 (20%)	16 (80%)	1.00	1.00	

1—reference group

*COR* crude odds ratio, *AOR* adjusted odds ratio, *CI* confidence interval

* Significant at P-value < 0.05

## Discussion

The overall quality of malaria microscopy in the assessed public health facility laboratories was 62.3%, which was considered to be poor. An ISO 15189 document requirement for quality and competence recommends above or equal to 80% [8]. This difference may be due to lack of training in malaria diagnosis and quality assurance, but was similar to the study conducted in Pakistan in which quality of malaria microscopy diagnosis was poor [9].

The current study revealed, 18 (60%) of health facility laboratories had in service trained laboratory professionals on malaria microscopy and a better quality of malaria microscopy diagnosis than those with no trained laboratory professionals [(AOR = 16 (1.3–1.96)]. A similar study conducted in health facilities in Oromia Regional State indicated 24% of health facilities participated laboratories in malaria microscopy diagnosis [10] while in Ethiopia 7 (6%) of health facilities participated in malaria microscopy diagnosis [11]. According to malaria laboratory diagnosis EQA scheme guidelines, laboratory professionals must have adequate training on malaria microscopy diagnosis and quality assurance to maintain quality implementation [7]. The study conducted in Hawassa health facility showed 50% of health facilities had trained laboratory professionals more than not trained [12]. This reflects a scarcity of training and refresher courses in malaria microscopy diagnosis. Low sensitivity and specificity on malaria parasites diagnosis indicated that there were many false negative results; which can lead to delayed treatment, development of serious complications and death.

This study showed that 80% and above of collected slides were good in staining in 7 (23.3%) of the health facility laboratories and had better quality in malaria microscopy diagnosis than health facility laboratories with poor blood film staining qualities. The difference was statistically significant [(AOR = 15, 95% CI (2.35, 8.61)] which was slightly better than 20% in the Democratic Republic of the Congo [13], but less than health facilities in Ethiopia at 31 (47%) [11].

Smearing and staining quality in the study area were known to be poor in routine laboratory settings, which has a great impact on patient results. Poor blood film preparation and staining generates artifacts commonly mistaken for malaria parasites, including bacteria, fungi, stain precipitation, dirty and cell debris. Normal blood components, such as platelets, also confound diagnosis. Improved training and higher quality of smear preparation and staining are required to reduce false readings.

The number of health facility laboratories with good detection and identification of *Plasmodium* species was 15 (50%) and 20 (66.7%), respectively. But the overall agreements of health facility laboratory professionals on detection and identification of plasmodium species with reference reader were 78 and 44.6% which was less than the national guideline recommendation [7]. It was also less than the study conducted in Africa 82% in parasite identification [14]. However, similar to detection with the study conducted in North Gondar (77%) [15].

Because of economic constraints, we did not assess all health facilities that perform malaria microscopic examination. Moreover, due to time limitation, the study could not evaluate the performance of health facilities regarding the quality of blood film preparation and staining procedures.

## Conclusion

In all assessed health facilities, malaria laboratory diagnosis was available but the overall quality of malaria microscopy diagnosis was poor. A significant gap was observed which could significantly impact on malaria microscopy quality services including untrained laboratory professionals on malaria microscopy diagnosis and quality assurance, poor blood film preparation, poor staining quality, poor parasite detection and identification.

## Abbreviations

QBC: quantitative buffy coat
WHO: World Health Organization
EQA: external quality assessment
ISO: International Standard Organization

## References

[1] WHO (2017). World malaria report.

[2] Cheesbrough M (2006). District laboratory practice in tropical countries.

[3] Raghuveer C, Rajeev A, Bhandari P (2008). Comparative study of peripheral blood smear, quantitative buffy coat and modified centrifuged blood smear in malaria diagnosis. Indian J Pathol Microbiol.

[4] Kettelhut M, Chiodini P, Edwards H, Moody A (2003). External quality assessment schemes raise standards: evidence from the UKNEQAS parasitology sub schemes. J Clin Pathol.

[5] WHO (2009). Malaria microscopy quality assurance manual.

[6] Annual East Wollega Zonal Health Department statistical report. June, 2014.

[7] Malaria laboratory diagnosis external quality assessment scheme guidelines. Ethiopian Federal Ministry of Health, September 2009.

[8] WHO-AFRO Laboratory Accreditation Assessment. Check list For Clinical and Public Health Laboratories- ISO 15189 document requirements for quality, and competence. 2012.

[9] Khan MA, Walley JD, Munir MA, Khan MA, Khobar NG, Tahir Z (2011). District level external quality assurance (EQA) of malaria microscopy in Pakistan: pilot implementation and feasibility. Malar J..

[10] Hailegiorgis B, Girma S, Melaku Z, Teshi T, Demeke L, Gebresellasie S (2010). Laboratory malaria diagnostic capacity in health facilities in five administrative zones of Oromia Regional State, Ethiopia. Trop Med Int Health..

[11] Abreha T, Alemayehu B, Tadesse Y, Gebresillassie S, Tadesse A (2014). Malaria diagnostic capacity in health facilities in Ethiopia. Malar J..

[12] Zeleke B, Admasu G, Getachew T, Kebede E, Belay G, Abraha A (2015). External quality assessment of malaria microscopy in Hawassa health facilities, Southern Ethiopia. Clin Med Res..

[13] Mukadi P, Gillet P, Lukuka A, Atua B, Sheshe N, Kanza A (2013). External quality assessment of Giemsa-stained blood film microscopy for the diagnosis of malaria and sleeping sickness in the Democratic Republic of the Congo. Bull World Health Organ.

[14] Frean J, Perovic O, Fensham V, McCarthy K, von Gottberg A, de Gouveia A (2012). External quality assessment of national public health laboratories in Africa, 2002–2009. Bull World Health Organ.

[15] Mitiku K, Mengistu G, Gelew B (2003). The reliability of blood film examination for malaria at the peripheral health unit. Ethiop J Health Dev..

